# Immediate Reversal of Hypoxemia With PFO Closure in Carcinoid Heart Disease and Platypnea-Orthodeoxia Syndrome

**DOI:** 10.1016/j.jaccas.2025.106659

**Published:** 2026-03-12

**Authors:** Jose F. Eduardo, Jose-Alejandro Ramirez-Penuela, Fabricio Malaguez Webber, Jesse Medina, Joao Braghiroli, Camilo Gomez

**Affiliations:** a University of Miami Miller School of Medicine, Miami, Florida, USA; b Department of Medicine, Universidad de Los Andes, Bogotá, Colombia; c Division of Cardiology, UHealth Jackson Health System, Miami, Florida, USA; d Department of Medicine, University of Miami & Jackson Health System, Miami, Florida, USA

**Keywords:** atrial septal defect, carcinoid heart disease, hemodynamics, hypoxemia, intracardiac echocardiography, occluder, patent foramen ovale, platypnea-orthodeoxia syndrome, right heart catheterization, tricuspid regurgitation

## Abstract

**Background:**

Intracardiac shunting via a patent foramen ovale (PFO) is an under-recognized cause of refractory hypoxemia.

**Case Summary:**

A 48-year-old woman with metastatic gastric neuroendocrine tumor involving the tricuspid valve presented with recurrent shortness of breath and severe hypoxemia refractory to supplemental oxygen. Computed tomography angiography ruled out pulmonary embolism. Transthoracic echocardiography with a bubble study demonstrated an interatrial communication suggesting a PFO. Right heart catheterization showed bidirectional shunting. Intracardiac echocardiography confirmed the presence of a large PFO. The patient underwent percutaneous PFO closure with an Amplatzer PFO Occluder, resulting in marked improvement in oxygenation and resolution of respiratory failure.

**Discussion:**

This case underscores the importance of considering intracardiac shunts in instances of unexplained refractory hypoxemia and demonstrates the efficacy of percutaneous PFO closure in treating this phenomenon.

**Take-Home Messages:**

Consider a PFO in persistent hypoxemia despite a negative pulmonary work-up. Timely percutaneous closure may lead to rapid, sustained improvement.

## Visual Summary

**Figure:**
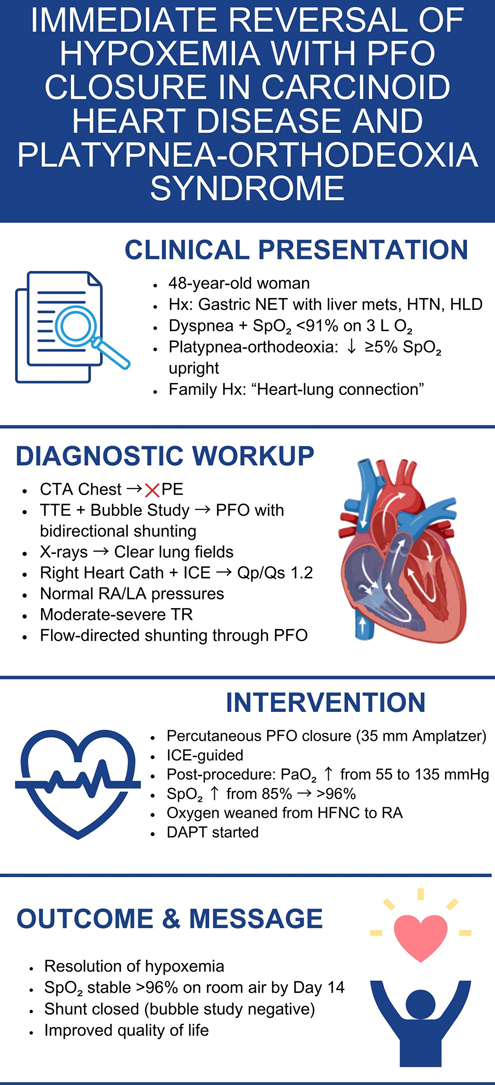

## History of Presentation

Our patient is a 48-year-old woman with a gastric neuroendocrine tumor with liver metastases, tricuspid valvar lesions, hypertension, and hyperlipidemia who presented to the emergency department with shortness of breath. She was tachypneic, with oxygen saturations in the low 90s on 3 L/min nasal cannula. Notably, her resting oxygen saturation dropped by >5% on moving from supine to upright, consistent with platypnea-orthodeoxia syndrome (POS). She had been hospitalized earlier that month for a similar episode of unexplained hypoxemia.

## Past Medical History

The patient had a history of neuroendocrine tumor with liver metastases, tricuspid valvar lesions, hypertension, and hyperlipidemia.

## Differential Diagnosis

**Figure 1:**
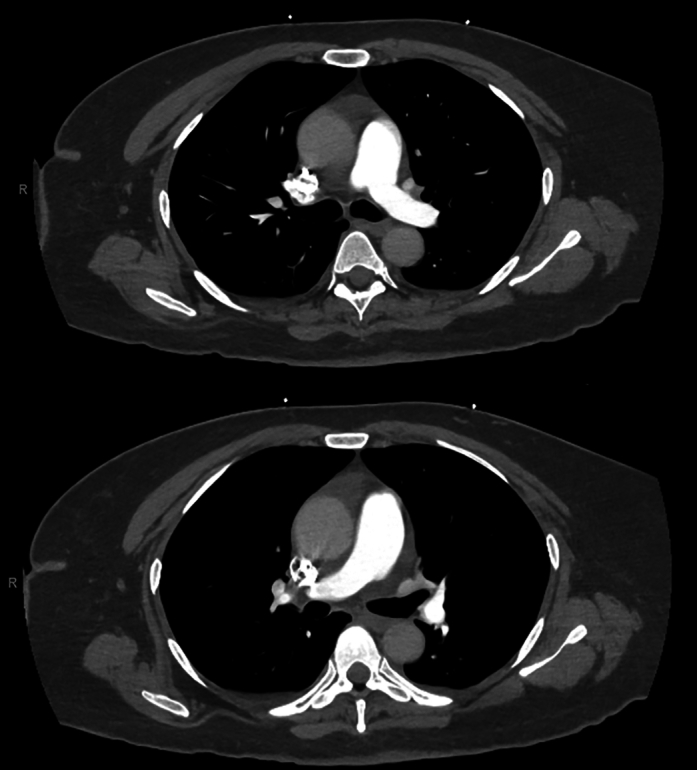
Computed Tomography Angiography of the Chest Axial computed tomography angiography image obtained on admission, demonstrating no evidence of pulmonary embolism.

**Figure 2:**
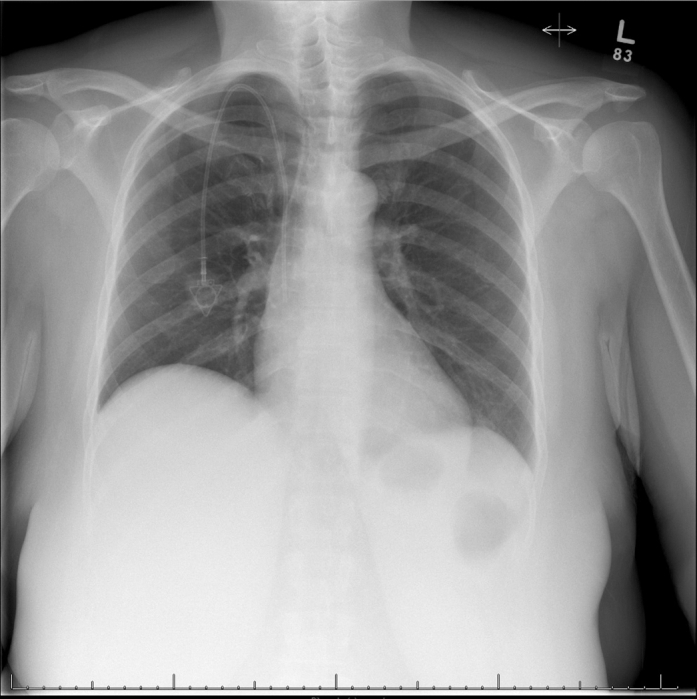
Baseline Anteroposterior Chest Radiograph Frontal chest radiograph on presentation, showing clear lung fields without consolidation or interstitial edema.

The differential diagnosis for hypoxemic respiratory failure included pulmonary embolism, pneumonia, cardiogenic pulmonary edema, and intracardiac shunting. Pulmonary embolism was promptly ruled out with computed tomography angiography (Figure 1), and chest radiography showed no edema or consolidation (Figure 2). Her recurrent hypoxemia refractory to supplemental oxygen, POS, and underlying neuroendocrine tumor pointed toward carcinoid-related intracardiac shunting.

## Investigations

**Figure 3:**
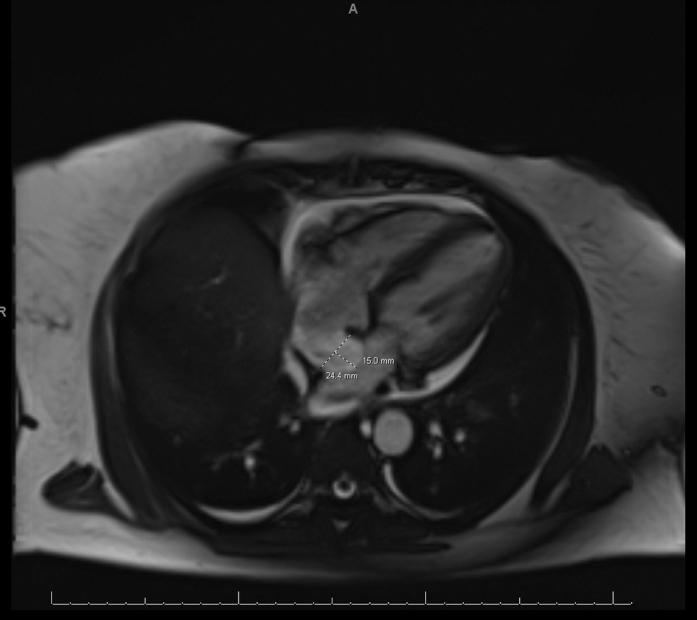
Cardiac Magnetic Resonance Imaging Cardiac magnetic resonance imaging demonstrating a 1.3-cm interatrial septal aneurysm (dashed line), supporting the presence of a structurally unstable septum predisposing to right-to-left shunting.

Initial arterial blood gas revealed primary respiratory alkalosis, with a pH of 7.47, a partial pressure of carbon dioxide of 24 mm Hg, and significant hypoxemia, with a partial pressure of oxygen 52 mm Hg. Serum chemistries demonstrated a potassium level of 2.7 mmol/L. Transthoracic echocardiography demonstrated a preserved left ventricular ejection fraction of 60% to 65%, severe tricuspid regurgitation, and an abnormal bubble study suggesting an interatrial communication (Videos 1 and 2). Given the patient's significant hypoxemia, she was considered high risk for transesophageal echocardiography. On hospital day 6, our team elected to pursue right heart catheterization (RHC) combined with intracardiac echocardiography (ICE) to safely assess an interatrial communication, measure intracardiac pressures, and perform a saturation run to evaluate intracardiac shunting (Video 3). RHC performed in the supine position on high supplemental oxygen confirmed a shunt with a pulmonary/systemic flow (Q_p_/Q_s_) ratio of 1.2, a preserved cardiac output of 5.26, and a cardiac index of 2.76, with right-sided filling pressures within normal limits (mean right atrial pressure 2 mm Hg; mean right ventricular end-diastolic pressure 3 mm Hg; mean pulmonary artery pressure 12 mm Hg; mean pulmonary capillary wedge pressure 4 mm Hg) (Table 1). This hemodynamic profile was suggestive of a bidirectional shunt. ICE imaging revealed a large patent foramen ovale (PFO) with bidirectional flow and moderate-to-severe tricuspid regurgitation. Cardiac magnetic resonance imaging confirmed a 1.3-cm interatrial septal aneurysm, with no large atrial septal defect (Figure 3). No left-sided carcinoid valvar involvement was present; both aortic and mitral valves appeared normal. These structural abnormalities further supported the PFO as the primary source of her severe hypoxemia.

**Table 1:** Right Heart Catheterization Findings

Oxygen Saturations	
Sampling Site	Oxygen Saturation
Right atrium	55.4%
Right ventricle	57.6%
Pulmonary artery	61.8%
Pulmonary capillary wedge	76.7%
Inferior vena cava	53.2%
Superior vena cava	58.4%
Aorta	94%
Q_p_/Q_s_ ratio	1.2

Hemodynamics	
Chamber/Vessel	Pressure
Right atrium A wave/V wave/mean	10/2/2 mm Hg
Right ventricle systolic/diastolic/mean	19/1/3 mm Hg
Pulmonary artery systolic/diastolic/mean	18/3/12 mm Hg
Pulmonary capillary wedge A wave/V wave/mean	6/6/4 mm Hg
Aorta systolic/diastolic/mean	111/71/85 mm Hg

Right heart catheterization demonstrated low right-sided oxygen saturations, normal intracardiac pressures, and a pulmonary/systemic flow (Q_p_/Q_s_) ratio of 1.2. In the setting of severe tricuspid regurgitation, these findings support a posture-dependent, flow-directed right-to-left shunt across the patent foramen ovale rather than a pressure-mediated intracardiac shunt.

## Management

Initial management focused on escalating supplemental oxygen, but the patient’s hypoxemia remained refractory despite high fraction of inspired oxygen (FiO_2_) and worsened with upright positioning, an important clinical clue to shunt physiology consistent with POS.

**Figure 4:**
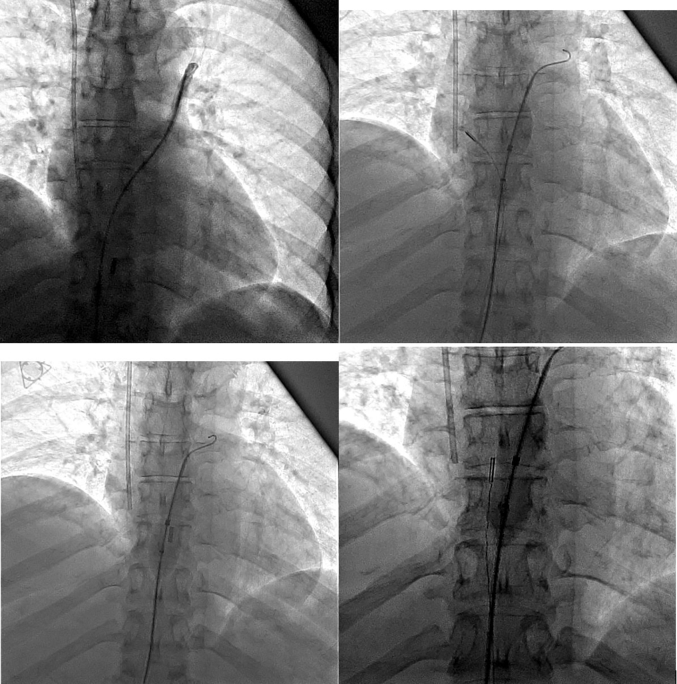
Intracardiac Echocardiography–Guided Right Heart Catheterization With Balloon Sizing of the Patent Foramen Ovale A 24-mm Amplatzer Sizing Balloon was inflated across the interatrial septum to occlude the defect and assess improvement in arterial oxygenation.

**Figure 5:**
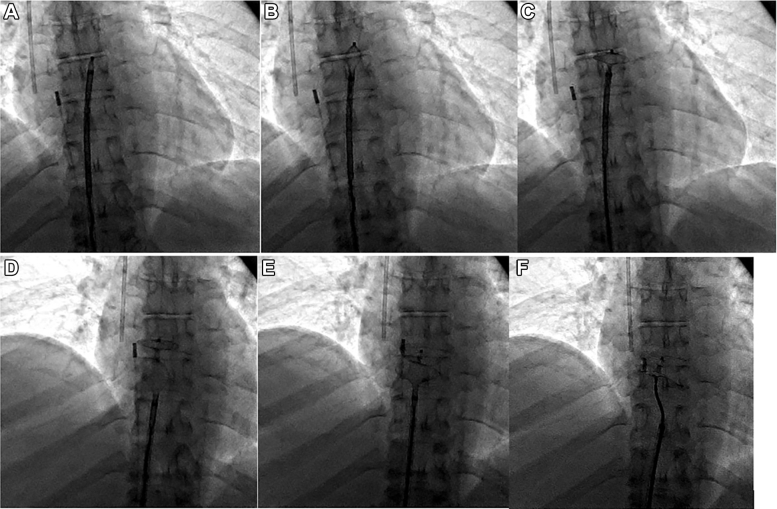
Sequential Fluoroscopic Images During Patent Foramen Ovale Occluder Deployment Sequential fluoroscopic images demonstrating deployment of a 35-mm Amplatzer PFO Occluder across the interatrial septum. (A) Advancement of the delivery sheath across the interatrial septum into the left atrium. (B) Partial deployment of the left atrial disc. (C) Full expansion of the left atrial disc with gentle retraction toward the interatrial septum. (D) Deployment of the right atrial disc, sandwiching the interatrial septum. (E) Assessment of device alignment and stability before device release. (F) Device configuration immediately after device release, confirming appropriate positioning. PFO = patent foramen ovale.

**Figure 6:**
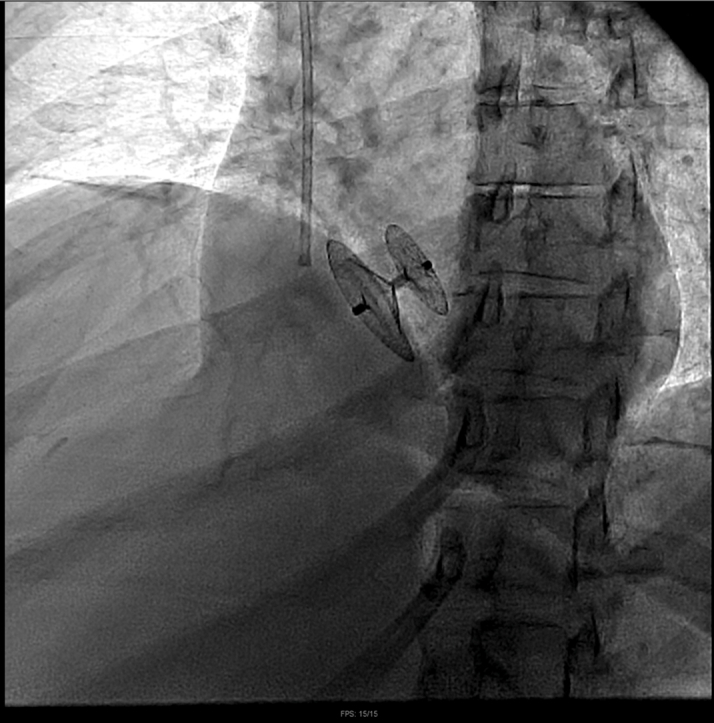
Lateral Fluoroscopic Projection During Patent Foramen Ovale Occluder Deployment Fluoroscopic lateral projection showing deployment of a 35-mm Amplatzer PFO Occluder across the interatrial septum, confirming appropriate orientation and positioning. PFO = patent foramen ovale.

**Figure 7:**
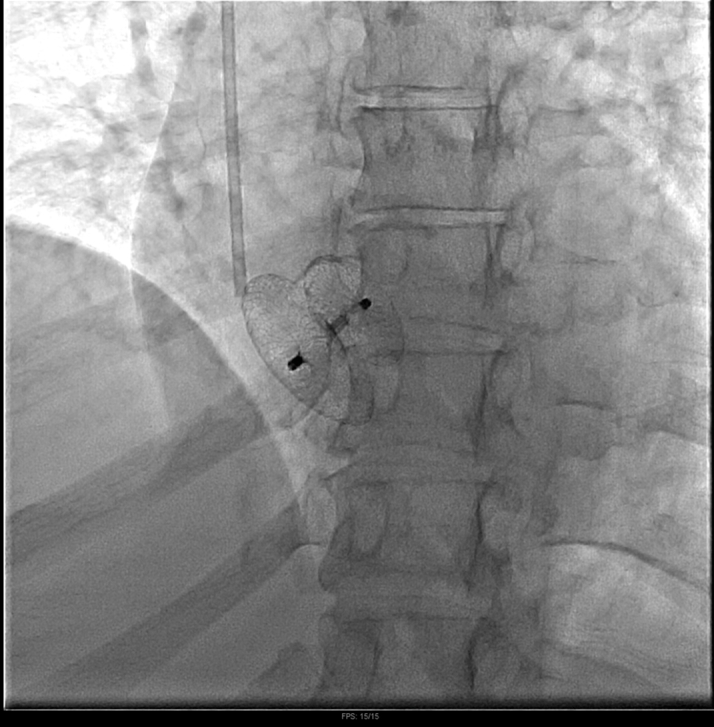
Frontal Fluoroscopic Projection Demonstrating Final Patent Foramen Ovale Occluder Position Fluoroscopic frontal projection demonstrating the Amplatzer PFO Occluder in a stable position, with no residual shunting confirmed by agitated saline injection. PFO = patent foramen ovale.

Her metastatic disease burden, normal right-sided pressures, and overall clinical status made her a poor candidate for immediate tricuspid valve intervention, so management prioritizing correcting the flow-driven right-to-left shunt was discussed. Given the patient's severe hypoxemia and inability to tolerate prolonged supine positioning during initial RHC, PFO closure was delayed until her respiratory status stabilized. She was scheduled for percutaneous PFO closure with ICE guidance on hospital day 8. After crossing the PFO, we used a 24-mm Amplatzer Sizing Balloon (Abbott) to occlude the interatrial defect and assess improvement in oxygenation, resulting in an immediate marked rise in partial pressure of oxygen, arterial (Pao_2_) from 55 mm Hg on 60% Fio_2_ to 146 mm Hg (Figure 4). Given this dramatic improvement, we proceeded with definitive closure using a 35-mm Amplatzer PFO Occluder (Abbott) (Figure 5). ICE images confirmed complete PFO closure and immediate resolution of interatrial shunting, with improvement in PaO_2_ from a preprocedural value of 55 mm Hg (on 60% FiO_2_) to approximately 135 mm Hg. ICE and fluoroscopic images confirmed successful device positioning (Figures 6 and 7, Videos 4 to 6).

## Outcome and Follow-Up

Our patient experienced an immediate and dramatic rise in oxygenation after percutaneous PFO closure and was started on dual antiplatelet therapy with aspirin and clopidogrel. Two days after PFO closure, she developed vasoplegic shock, consistent with carcinoid crisis, a known complication of metastatic neuroendocrine tumors, but without hemodynamic evidence of acute right ventricular failure; right-sided filling pressures remained normal; and follow-up echocardiography demonstrated preserved right ventricular systolic function without new volume overload. She recovered with supportive care.

Although not directly related to PFO closure, the event highlights the systemic manifestations of metastatic carcinoid disease and the importance of co-ordinated multidisciplinary follow-up. Her oxygen saturation subsequently stabilized at 95% to 100% on progressively lower support, weaning to room air with peripheral oxygen saturation >96% by hospital day 14. Outpatient follow-up with interventional cardiology and oncology was arranged.

## Discussion

POS in this case underscores a flow-driven shunt physiology rather than pressure overload: severe tricuspid regurgitation from carcinoid involvement generates an eccentric jet that is directed toward and through a PFO when the patient is upright. Upright posture reduces left atrial preload while augmenting right atrial volume overload, thereby increasing the right-to-left gradient and precipitating marked positional hypoxemia. Recognition of this dynamic shunt was pivotal: confirming a >5% drop on positional oximetry and visualizing bidirectional flow on upright bubble study allowed tailored monitoring and selection of ICE for supine device guidance. ICE safely provided real-time septal visualization and precise device guidance without the need for transesophageal echocardiography. Our patient's profound hypoxemia increased her risk of even mild respiratory drive suppression associated with sedation. This is an important consideration in high-risk patients with refractory hypoxemia.^1^ This focused understanding of shunt mechanics informed our clinical decision making.

From a management standpoint, distinguishing flow-driven from pressure-driven shunting was essential in determining the safety of PFO closure. In patients with pulmonary hypertension or elevated right-sided pressures, an interatrial communication may provide necessary decompression of the right ventricle, making closure relatively contraindicated. In our patient, right-sided pressures and mean pulmonary artery pressure were normal, and the modest Q_p_/Q_s_ ratio of 1.2 indicated no large left-to-right shunt or fixed pressure overload. During balloon test occlusion, Pao_2_ rose dramatically without increases in right-sided pressures, confirming that the PFO was not serving as a pressure-relief pathway. In this context, the PFO functioned not as a pressure-relief “pop-off valve” for a failing right ventricle but as a conduit for flow-directed venous streaming, which became pathologic primarily in the upright position.^2^

The Q_p_/Q_s_ ratio of 1.2 obtained during supine catheterization reflects a small net left-to-right shunt with normal pulmonary pressures and does not exclude significant right-to-left flow in the upright position. In POS, posture-dependent changes in atrial geometry and venous streaming can produce transient right-to-left shunting, which is not captured by a single supine measurement.^2^

This case highlights the importance of considering an intracardiac shunt, particularly a PFO, in unexplained hypoxemia that remains refractory to increasing Fio_2_, a hallmark diagnostic clue of shunt physiology. Although PFOs are prevalent in approximately 25% of the adult population and are often asymptomatic, they become clinically significant in the setting of carcinoid heart disease, where right-to-left shunting can be precipitated despite normal pulmonary pressures.^3^^,^^4^ Prospective echocardiographic studies demonstrate a markedly higher prevalence of PFO in this population, identified in 41% of patients with carcinoid syndrome and up to 59% of those with established carcinoid heart disease, roughly twice the rate seen in the general population, providing an anatomic predisposition to severe positional hypoxemia.^5^^,^^6^

In our case, moderate-severe tricuspid regurgitation due to metastatic carcinoid heart disease likely contributed to right atrial volume overload and increased shunt flow.^7^ Carcinoid heart disease, characterized by right-sided valvar lesions, can induce functional hemodynamic shifts that increase the likelihood of PFO opening and pathologic shunting, even in the absence of elevated mean pulmonary pressures. This pathophysiologic mechanism aligns with prior case reports. Upright posture, right atrial dilation, and prominent Eustachian valves may all contribute to redirection of venous return across the interatrial septum, leading to positional hypoxemia despite preserved intracardiac pressures.^5^

Percutaneous closure of a PFO has shown favorable outcomes in selected patients with POS and refractory hypoxemia, with recent series demonstrating immediate and sustained improvements in oxygenation and symptom burden postclosure.^3^^,^^8^ In our patient, oxygenation improved dramatically from 55 to 135 mm Hg postprocedure, mirroring outcomes described in these reports.

Although stroke prevention remains the primary indication for PFO closure, this case builds on a growing literature in oncology and carcinoid heart disease, where targeted closure in patients with PFO can rapidly reverse hypoxemia from right-to-left shunting and may limit left-heart exposure to vasoactive, profibrotic substances bypassing pulmonary filtration.^3^^,^^6^^,^^9^ It underscores the importance of maintaining a high index of suspicion for intracardiac shunts in complex patients and using a systematic work-up, including transthoracic echocardiography with a bubble study, transesophageal echocardiography, RHC, and ICE, to identify reversible causes of hypoxia. By expanding cardio-oncology to address structural complications, such as PFO-mediated shunting, this multidisciplinary approach, which combines oncologic care with interventional cardiology, offers a road map to improve quality of life in patients with limited systemic treatment options.^10^

## Conclusions

In patients with unexplained hypoxemic respiratory failure, especially in the context of recurrent hospital stays and negative pulmonary work-up, clinicians should consider an intracardiac shunt such as a PFO. A multidisciplinary approach incorporating advanced imaging and percutaneous intervention can lead to significant symptomatic and functional improvement. Clinicians should maintain a high level of suspicion for POS in any patient with carcinoid heart disease with new or worsening hypoxemia, particularly when right-sided valvar lesions are evident.^5^

**Visual Summary:**
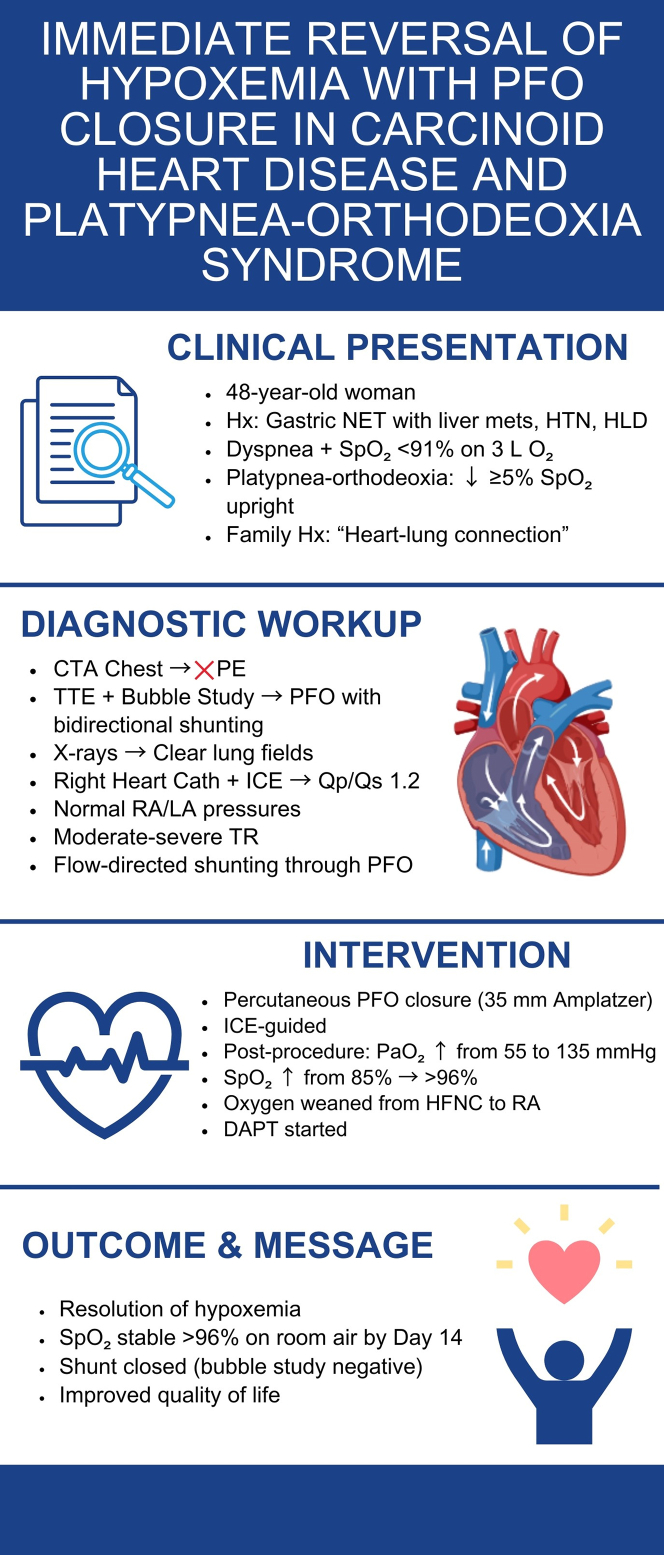
Percutaneous PFO Closure for Platypnea-Orthodeoxia Syndrome Visual summary of (A) Clinical presentation, (B) diagnostic work-up, (C) intervention, and (D) outcomes. After ICE–guided deployment of a 35-mm Amplatzer device, partial pressure of oxygen, arterial (Pao_2_) improved from 55 to 135 mm Hg, Spo_2_ rose from 85% to >96% on room air, and flow-directed shunting through the PFO was abolished. DAPT was initiated, and the patient’s quality of life markedly improved by hospital day 14. Cath = catheterization; CTA = computed tomography angiography; DAPT = dual antiplatelet therapy; HFNC = high-flow nasal cannula; HLD = hyperlipidemia; HTN = hypertension; ICE = intracardiac echocardiography; LA = left atrium; NET = neuroendocrine tumor; Pao_2_ = partial pressure of oxygen, arterial; PE = pulmonary embolism; PFO = patent foramen ovale; Qp/Qs = pulmonary/systemic flow; RA = right atrium; Spo_2_ = peripheral oxygen saturation; TR = tricuspid regurgitation; TTE = transthoracic echocardiography.

## Funding Support and Author Disclosures

The authors have reported that they have no relationships relevant to the contents of this paper to disclose.

**Take-Home Messages**

• In carcinoid heart disease, flow-driven right-to-left shunting through a patent foramen ovale may occur even in the absence of elevated right atrial pressure.
• Upright posture and valvar jet dynamics can facilitate significant intracardiac shunting, which contributes to hypoxemia.
• Percutaneous closure should be considered not only as a palliative intervention but also as a therapeutic strategy to improve hypoxemia, relieve dyspnea, enhance cardiopulmonary pressures and flow, and ultimately improve quality of life in these patients.

## Appendix

Video 1 Bubble Study Demonstrating Intracardiac Shunting Preclosure Agitated saline contrast transthoracic echocardiography demonstrates immediate left atrial opacification after right atrial filling, confirming right-to-left intracardiac shunting through the patent foramen ovale, consistent with platypnea-orthodeoxia syndrome.

Video 2 Severe Tricuspid Regurgitation Before Intervention Color Doppler transthoracic echocardiography demonstrates torrential tricuspid regurgitation with right atrial volume overload and eccentric jet formation, consistent with carcinoid valvular involvement.

Video 3 Intracardiac Echocardiography During Right Heart Catheterization ICE imaging demonstrates visualization of the interatrial septum with bidirectional flow across a large patent foramen ovale and Doppler assessment of associated flow-directed shunting during diagnostic catheterization.

Video 4 Percutaneous PFO Closure: Full Procedural Sequence Fluoroscopic and intracardiac echocardiography guidance demonstrates balloon sizing of the patent foramen ovale, followed by deployment of a 35-mm Amplatzer PFO Occluder and confirmation of stable device positioning.

Video 5 Postclosure Intracardiac Echocardiography ICE imaging confirms successful occluder deployment with the absence of residual interatrial flow after definitive PFO closure.

Video 6 Postclosure Agitated Saline Bubble Study Repeat contrast echocardiography demonstrates no left atrial opacification after right atrial injection, confirming complete elimination of intracardiac right-to-left shunting after PFO closure.
